# Optimization of the Processing Conditions for the Production of a Gluten-Free Bread from Sour Cassava Starch (*Manihot esculenta*) and Some Legumes (*Arachis hypogaea*, *Vigna unguiculata*, and *Glycine max*)

**DOI:** 10.3390/foods12173180

**Published:** 2023-08-24

**Authors:** Marie Madeleine Nanga Ndjang, Julie Mathilde Klang, Bilkissou Njapndounke, Marius Edith Kouam Foko, Jean Roger Dongmo, Michael Hermann Kengne Kamdem, Jordan Lembe Tonga, Edwin Mpho Mmutlane, Derek Tantoh Ndinteh, Eugenie Kayitesi, François Ngoufack Zambou

**Affiliations:** 1 Research Unit of Biochemistry of Medicinal Plants, Food Sciences and Nutrition, Department of Biochemistry, Faculty of Science, University of Dschang, Dschang P.O. Box 67, Cameroon; ndjang31@gmail.com (M.M.N.N.); jrdongmo@gmail.com (J.R.D.); fzambou@gmail.com (F.N.Z.); 2 Centre for Natural Products Research, Department of Chemical Sciences, University of Johannesburg, Doornfontein Campus, P.O. Box 17011, Johannesburg 2028, South Africa; michaelhermannkamdem@gmail.com (M.H.K.K.); jtongalembe2065@gmail.com (J.L.T.); dndinteh@uj.com (D.T.N.); 3 Laboratory of Microbiology, Department of Microbiology, Faculty of Science, University of Yaoundé I, Yaoundé P.O. Box 812, Cameroon; njapndounkebilkissou123@yahoo.com; 4 Department of Physiological Sciences and Biochemistry, Faculty of Medicine and Pharmaceutical Sciences, University of Dschang, Dschang P.O. Box 67, Cameroon; fokokouam@gmail.com; 5 Research Center for Synthesis and Catalysis, Department of Chemical Sciences, University of Johannesburg, Kingsway Campus, Auckland Park, Johannesburg 2008, South Africa; edwinm@uj.ac.za; 6 Department of Food and Consumer Science, University of Pretoria, Private Bag 20, Hatfield, Pretoria 0028, South Africa; eugenie.kayitesi@up.ac.za

**Keywords:** breadmaking, cowpea, I-optimal design, peanut, sour cassava starch, soybean

## Abstract

Background: Sour cassava starch is used as an alternative to wheat flour in breadmaking. However, its nutritional and technological properties are limited. To remedy this, the use of legumes has proved to be very successful. Thus, the present study aimed to determine the optimal condition for the production of bread made from sour cassava starch, peanut, cowpea and soybean flour. Methods: The I-optimal design was employed to obtain an optimal proportion of the mixture with the variables sour cassava starch, cowpea, soy and peanut flour. The responses evaluated were overall acceptability, specific volume and protein content. Results: It resulted that the incorporation of sour cassava starch positively influenced the volume but negatively influenced the protein content and overall acceptability. While the addition of legumes increased protein content and overall consumer acceptability, the specific volume was reduced. The optimal proportions of sour cassava starch, cowpea, soybean and peanut flour were 64.11%, 18.92%, 0% and 16.96%, respectively. Under this condition, it led to a desirability of 1, specific volume of 1.35, overall acceptability of 6.13, protein content of 9.72%, carbohydrate content of 67.89%, fat content of 9.39%, fiber content of 2.10% and ash content of 1.04%. Conclusions: The findings suggest that cowpea and peanut can be used for the improvement of the technological, nutritional and sensory properties of sour cassava starch bread and thus increase its consumption and application in the food processing industry.

## 1. Introduction

Cassava (*Manihot esculenta Crantz*, *Euphorbiaceae*), also known as yuca, manioc, mandioc, mandioca or tapioca, is a vegetatively propagated perennial shrub grown in the lowlands of the tropics for its starchy tubers with thickened roots [1]. It is the world’s second-largest producer of starches after maize—the global production is estimated to be 292 Mt of fresh tubers, or 35% of dry weight, and it can be grown at a significantly lower cost [2,3]. In Cameroon, cassava is the second source of starch after yam in terms of production, estimated at 4.858329 million tons per year [3]. Cassava starch has many functional properties which can be exploited in many domains such as food applications. Such is the case in breadmaking, where cassava starch is used as a substitute for wheat flour. Indeed, several problems are associated with the use of wheat flour alone in breadmaking, including gluten intolerance and the unavailability of this commodity in several countries that depend entirely on imports. This destabilizes food security, and to remedy this situation, the use of locally available flour such as cassava is proving to be very promising.

The breadmaking ability of cassava starch is conferred by a number of treatments such as 30-day fermentation and solar drying [4]. The resultant product is sour starch which can be used as a wheat alternative in breadmaking. Cassava starch acquired during fermentation and subsequent solar drying has better breadmaking ability associated with the expansion of water vapor bubbles retained by the network of starch molecules alone [5]. Nonetheless, this fermentation step does not occur during the breadmaking process using wheat flour because the dough only swells during cooking. However, once the expansion limit is reached, an undesirable contraction of the dough occurs, resulting in poor-quality bread. In addition, cassava starch is poor in proteins, lipids and minerals that are essential for the proper functioning of the body. Proteins provide amino acids and carbon skeleton for the metabolic needs of the body [6]; lipids form cell membrane structures and thus control the movement of substances in and out of cells. Also, minerals have key roles in our body, from building strong bones to transmitting nerve impulses. However, minerals such as aluminum (Al) and cobalt (Cd) are dangerous at high concentrations [7]. Hence, for a healthy and lengthy life, these nutrients have to be provided in an adequate amount. Therefore, the incorporation of other ingredients such as protein and mineral sources to the sour cassava starch would allow us to obtain better quality breads. Also, the combination of proteins and polysaccharides results in films with excellent swelling properties because they constitute barriers to oxygen and carbon dioxide, thus improving the structural integrity of products (bread) [8]. Moreover, the combination of sour cassava starch and legumes will compensate for the protein and mineral deficit and technological quality of the resulting bread.

Legumes are excellent sources of proteins and minerals; they have rheological properties that can be exploited in breadmaking. Hence, their association with sour cassava starch can improve the nutritional and technological quality of its bread. In this context, Ref. [9] analyzed the influence of a protein concentrate used as a fat substitute on the quality of cheese bread. Ref. [10] studied the changes in the physical properties of snacks based on a mixture of sour cassava starch and quinoa flour; Ref. [11] studied the physical properties of extruded snacks based on a mixture of sour cassava starch and flaxseed meal; Ref. [12] developed a cheese bread enriched with biofortified whole cowpea flour. However, to the best of our knowledge, little or no work has been reported on the incorporation of cowpea, soybean and peanut in the breadmaking process with sour cassava starch. Hence, the objective of this work was to improve the nutritional and technological quality of sour cassava starch bread by the incorporation of cowpea, soybean and peanut flours.

## 2. Materials and Methods

### 2.1. Materials

The plant material consisted of peanut, soybean and cowpea seeds, collected at the local market in Dschang, and cassava tubers, collected at a farmer’s field in Bertoua. Baking ingredients consisted of composite flour, water and salt. The salt (white Diamant), provided by SOCAPURSEL (Douala, Cameroon), was purchased at the local supermarket. The chemicals reagents included ammonium sulfate ((NH_4_)_2_SO_4_), sulfuric acid (H_2_SO_4_), distilled water (H_2_O), boric acid (H_3_BO_3_), color indicators (bromocresol green (C_21_H_14_Br_4_O_5_S) and methyl red (C_15_H_15_N_3_O_2_)), sodium hydroxide (NaOH), hydrogen chloride (HCL), hexane (C_6_H_14_), nitric acid (HNO_3_), deionized water (H_2_O), and standards solutions of aluminum (Al), boron (B), barium (Ba), calcium (Ca), cadmium (Cd), chromium (Cr), cobalt (Co), copper (Cu), iron (Fe), potassium (K), lithium (Li), magnesium (Mg), manganese (Mn), molybdenum (Mo), sodium (Na), nickel (Ni), phosphorus (P), silicon (Si) and zinc (Zn). All the chemicals and reagents used in this study were of analytical grade and were purchased from Sigma-Aldrich Pty.Ltd. (Johannesburg, South Africa)

### 2.2. Sampling

The cassava starch was wet-extracted in a traditional production unit (*Société coopérative simplifiée des transformations de manioc* (SCSTM)). Here, the starch was extracted, fermented for 30 days and exposed to light rays for around 24 h. The soybean and cowpea grains were soaked for 12 h and then dried in an oven at 45 °C for about 24 h, ground and sieved (160 µm) Aleem et al. [13]. The protocol described by Salve and Arya. Ref. [14] was used to obtain peanut flour; briefly, groundnut seeds were roasted and then ground into flour.

### 2.3. Characterization of the Starch and the Different Flours

#### 2.3.1. Proximate Composition

The moisture content was determined according to the standard method [15], which consists of eliminating water from a product by drying it in the oven at 105 °C until it reaches a constant weight. Ash content was determined according to AACC 44-40-01 [16]; here, the samples were incinerated at 550 °C in an oxidizing atmosphere until the inorganic residue of constant mass was obtained. For the fiber content, it was determined using the AOAC 985.29 [17] method, which is based on the weight loss of the sample after incineration. Total lipids were extracted with Soxhlet according to the Russian method described by Bourely [18], which has to do with the differential solubility of lipids in organic solvents (hexane or petroleum ether). It was executed at a high temperature for about 8 h, after which the solvent remaining on the sample was removed by drying it in an oven. The determination of protein content was performed using the improved Kjeldahl method for nitrate-free samples as described by AACC 46-11 [19], which is based on the transformation of organic nitrogen into ammoniacal nitrogen, by mineralization with concentrated sulfuric acid. The total carbohydrate content was determined by the difference method AOAC [20], where the weights of ash, protein and fat were subtracted from 100.

#### 2.3.2. Mineral Profile Using Inductively Coupled Plasma Optical Emission Spectrometry (ICP-OES)

Here, 1 g of each sample was weighed into digestion vessels and mixed with 10 mL of concentrated nitric acid (HNO_3_) using a modified method of Biata et al. [21]. After that, the mixture was later heated in a fume cupboard. Before analysis, the digest was cooled, transferred to a 100 mL volumetric flask, diluted to the mark with deionized water and then filtered (0.45 m). ICP-OES standards solutions of aluminum (Al), boron (B), barium (Ba), calcium (Ca), cadmium (Cd), chromium (Cr), cobalt (Co), copper (Cu), iron (Fe), potassium (K), lithium (Li), magnesium (Mg), manganese (Mn), molybdenum (Mo), sodium (Na), nickel (Ni), phosphorus (P), silicon (Si) and zinc (Zn) were prepared. All extractions were performed in triplicate, and samples were analyzed in triplicate as well. ICP-OES equipment (iCAP 6500 Duo, Thermo Scientific, Basingstoke, UK) was used with the following parameters and conditions: RF power (emission intensity)—1150 W; nebulizer—0.75 L/min gas flow, 0.5 L/min auxiliary gas flow, 12 L/min coolant gas flow; purge gas flow—boost; nebulizer type—cross-flow.

#### 2.3.3. Determination of Anti-Nutrient Content

Total oxalate content was determined by hot titration of an aliquot of the sample extract with a 0.01 M KMnO_44_ solution following AOAC [22]. The tannin content of the various flours was assessed using the method described by Bainbridge et al. [23]. Here, the determination was performed spectrophotometrically at 500 nm by the difference in absorbance. The standard curve was prepared using tannic acid and the tannin content was expressed as mg tannic acid equivalents/100 g of dry matter. The phytate content was determined by titration of an aliquot of the sample extract with a standard solution of Fecl_3_ AOAC [24].

### 2.4. Mixture Design

The “I-optimal” mixing design was used to obtain the optimum conditions for bread formulation by optimizing the level of independent variables. I-optimal was chosen in this study because it minimizes the average variance of prediction and therefore seems more appropriate for mixture experiments [25]. The factors and experimental domains were chosen based on their high protein content and the preliminary tests; the amounts of sour starch (X1. 45–95% g/g), peanut (X2. 0–40% g/g), cowpea (X3. 0–28% g/g) and soybean flour (X4. 0–38% g/g) and other ingredients, namely water and salt, were kept constant. The responses were overall acceptability, specific volume and protein content. All experiments were performed in duplicate, and the resulting experimental matrix is presented in Table 4. The fitted response values are represented by the linear (Y1), quadratic (Y2) and special cubic (Y3) models:

Y = b1X1 + b2X2 + b3X3 + b4X4(1)

Y = b1X1 + b2X2 + b3X3 + b4X4 + b1b2X1X2 + b1b3X1X3 + b1b4X1X4 + b2b3X2X3(2)

Y = b1X1 + b2X2 + b3X3 + b4X4 +b1b2X1X2 + b1b3X1X3 + b1b4X1X4 + b2b3X2X3 + b1b2b3X1X2X3(3)

where Y is the expected response; b1, b2, b3 and b4 are the coefficients.

### 2.5. Model Validation

There are six indices for assessing model suitability. A *p*-value less than 0.05 indicates that the factors or the effects of their interactions have a significant influence on the response. The R^2^ reveals whether or not the model is appropriate, and the closer it is to 1, the more appropriate the model is. The adjusted R^2^ (R^2^ Adj) is used to calculate all variations around the model mean. The predicted R^2^ (R^2^ Pred) is adopted to estimate the extent to which the model predicts the value of the response. PRESS (predicted residual error sum of squares) is used to judge the model’s suitability; the smaller it is, the more appropriate the model is. In addition, it must have a correct “lack of fit”. Indeed, the “lack of fit” is correct when its *p*-value is not significant [26] (Table 5).

### 2.6. Bread Production and Evaluation of the Responses

#### 2.6.1. Bread Production

The method developed by Alvarado et al. [4] was used. The different formulations were mixed with salt and water in a mixer at low speed (165 rpm) for 1 min and then at medium speed (300 rpm) for 2 min. The dough obtained was shaped and the dough pieces were baked for 30 min at 250 °C before cooling at room temperature for 2 h.

#### 2.6.2. Overall Acceptability

The hedonic test was conducted using the 9-point score method as described by Koppel [27]. The bread samples were simultaneously presented to the tasters (60 naive panelists) who were asked to fill out a form reporting their appreciation of the bread on a scale ranging from 1 (extremely unpleasant) to 9 (extremely pleasant) for the specific descriptors (appearance, odor, texture, taste, crispness). The overall acceptability was obtained by averaging all specific descriptors.

#### 2.6.3. Specific Volume

The loaves’ specific volume was determined using the repeated displacement method of AACC International (Approved Methods10-05.01) [28].

#### 2.6.4. Protein Content

The determination of protein content was performed using the Kjeldahl method (AACC 46-11) [19], which is based on the transformation of organic nitrogen into ammoniacal nitrogen by mineralization with concentrated sulfuric acid.

### 2.7. Multiple Optimizations of the Responses

Optimization of the different responses was carried out according to the following conditions: the overall acceptability was maximized, the specific volume was targeted to be 2.6 g/cm^3^ according to the benchmark bread, and the protein content was in the range of 1.24–16.56 according to the data obtained from the different trials. A compromise was then made between the different optimal conditions obtained for each response, and an optimal formulation was chosen.

### 2.8. Evaluation of the Pasting Properties of Sour Cassava Starch, Cowpea Flour, Peanut Powder and Optimum Flour

The viscous behavior of starch at various fermentation times and varieties was measured using a Rapid Visco Analyzer model RVA-4 Series (Newport Scientific, Warriewood, Australia) as described by Sánchez et al. [29]. A mass of 2.5 g (dry basis) of flour was dispersed in 22.5 g of water. The following temperature profile was used to measure viscosity: holding at 50 °C for 1 min, heating from 50 °C to 90 °C at 6 °C·min^−1^, holding at 90 °C for 5 min, and then cooling down to 50 °C at 6 °C·min^−1^ with continuous stirring at 960 rpm for 10 s and then at 160 rpm for the rest of the experiment. The visco-amylogram yielded the following seven parameters: peak viscosity, minimum viscosity (MV), breakdown (BD), final viscosity (FV), setback (SB), peak time (PT) and pasting temperature (PTC). The analyses were performed in triplicate and mean values were calculated.

### 2.9. Comparative Study of the Optimal Bread with Cheese Bread

Cheese bread is a traditional Colombian braid made with sour cassava starch and grated cheese known as *pan de quezo or pao de queijo* or *Yuca bread*. It is also very common in Brazil and Latin America where it originates. Since it is in this form that sour cassava starch bread is known and consumed, we took it as our benchmark bread in this study. For this part, we compare the proximate composition, sensory parameters and textures of the two breads.

#### 2.9.1. Evaluation of the Proximate Composition

The proximate composition was conducted according to standard methods as described in Section 2.3.1 and Section 2.3.2.

#### 2.9.2. Evaluation of the Sensory Parameters

These parameters were determined following the protocol explained in Section 2.6.2.

#### 2.9.3. Evaluation of the Textural Properties

Texture profile analysis (TPA) of sourdough bread crumbs was performed using a texture analyzer (Universal Texture Machine, LR 5K, Lloyd Instruments Ltd.,Bognore Regis, UK) equipped with an 80 mm diameter cylindrical probe. TPA was performed by two sequential compressions (speed 50 mm/min; compression 50%; load 1 KN). The obtained profile was used to calculate hardness, cohesiveness, consistency, elasticity and plasticity using Nexygen software 4.1 (Lloyd material testing, West Sussex, UK). The resulting profile was used to calculate hardness, cohesiveness, elasticity, gumminess, chewiness, stickiness and stiffness using Nexygen software 4.1 (Lloyd material testing, West Sussex, UK) following Shobharani et al. [30]. The test was carried out in triplicate.

### 2.10. Statistical Analysis

The mixture trials were generated using Design-Expert version 11.0 software. Excel version 2013 software was used to express the results of the analyses performed in triplicate as means plus or minus standard deviations, and analysis of variance (ANOVA) was used for statistical analysis using SPSS version 23 software.

## 3. Results and Discussion

### 3.1. Proximate Composition of the Different Flours

In order to assess the nutritional contribution of legume flour to sour cassava starch, a bromatological analysis was carried out on sour cassava starch, peanut flour, cowpea flour and soy flour. Table 1 and Table 2 show the chemical composition of the different flours. It appears that the protein, lipid, carbohydrate, water, fiber and mineral contents differ significantly at *p* < 0.05 between the different flours. Also, cowpea flour, soy flour and peanut flour are richer than sour starch in protein, lipids, fiber and minerals. This is because legumes are of higher nutritional quality than cassava starch [31]. They also have a lower moisture content, characteristic of good preservation [32]. The contents of heavy metals such as Cr, Cu, Ni, Al, Fe and Cd, which can be dangerous to the proper functioning of the body, are low or even absent (Table 2) and therefore cannot harm the consumer [33]. Table 3 shows the phytate, tannin and oxalate composition of the various flours. It should be noted that the anti-nutrient content gives an idea of the toxicity and the bioavailability of minerals in legume flours. We can observe that the anti-nutrient content differs significantly at *p* < 0.05 between the different flours. Also, it appears that more than 50% of the initial content of anti-nutrient has been reduced as a result of the various treatments applied [34]. Indeed, the decrease in phytate, oxalates and tannin content resulted in an increase in the amount of total Fe, Zn and Ca [35]. Thus, the reduction in the antinutrients enhanced the bioavailability of minerals (Fe, Zn and Ca).

### 3.2. Mixture Design

#### 3.2.1. Presentation of Different Factors and Responses

The “I-optimal design” allowed us to perform 20 trials. It appears that the overall acceptability ranged from 3.96 to 6.13, the specific volume from 0.4 to 2.64 g/cm^3^ and the protein content from 1.24 to 16.56 g/100 g (Table 4).

#### 3.2.2. Mathematical Model

Design-Expert 11.0.5.0 was used in this study to perform regressions, graphical analyses and analysis of variance (ANOVA). Linear, two-factor interaction (2FI), quadratic and cubic models were examined and compared to select the best model for the experiment (Table 5). The linear, quadratic and special cubic models appear to be the best suited to the analyses. The linear model proved to be the best model for the protein content, the quadratic model for the specific volume and the special cubic model for the overall acceptability. The cubic model was aliased which means that the effect of each variable that cause different signals become indistinguishable. The coefficient of determination indicates the extent to which the total variability of the variable response is explained by the fitted model, a value greater than 0.75 (75%) being preferable. In addition, lack of fit is a measure of a model’s weakness in representing the data for the experimental region. Therefore, an appropriate model is one in which the lack of fit is not significant [36]. The results show that the models fitted for the response were appropriate, with an R^2^ greater than 75% and no significant lack of fit (*p* > 0.05). Consequently, the concentration of these responses explains much of their variability.

#### 3.2.3. Effect of Components, Model Fitness, Model Testing and Responses Surface Analysis of the Overall Acceptability

**Figure 1:**
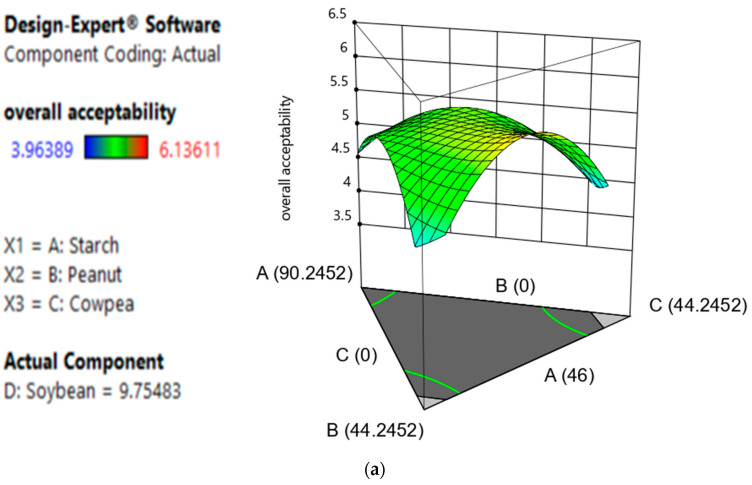

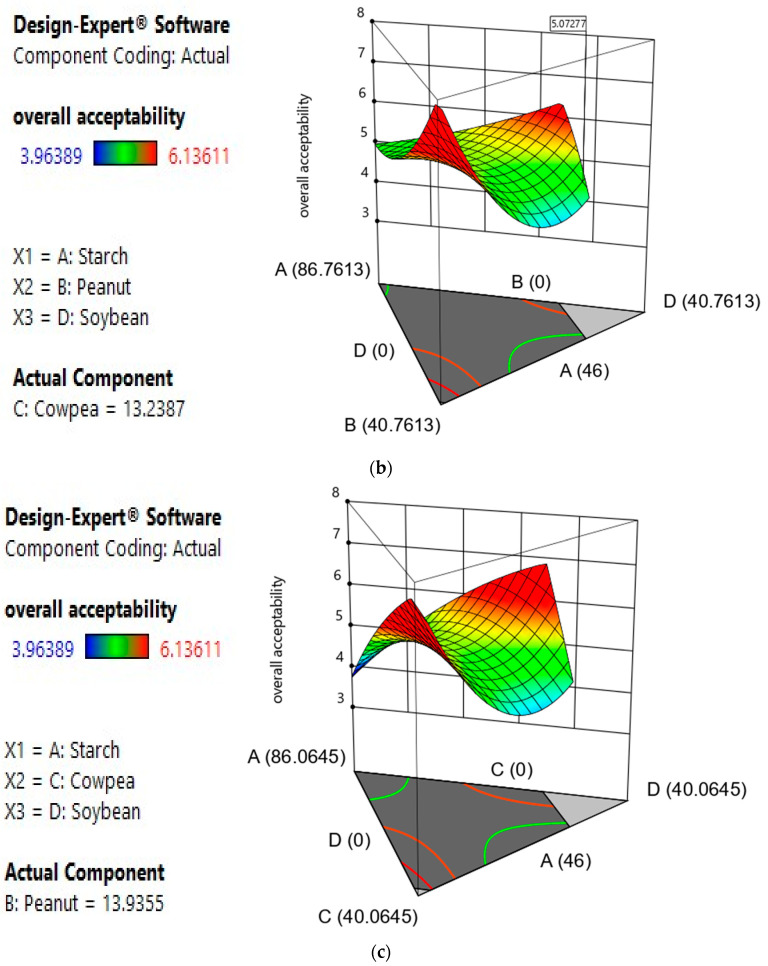

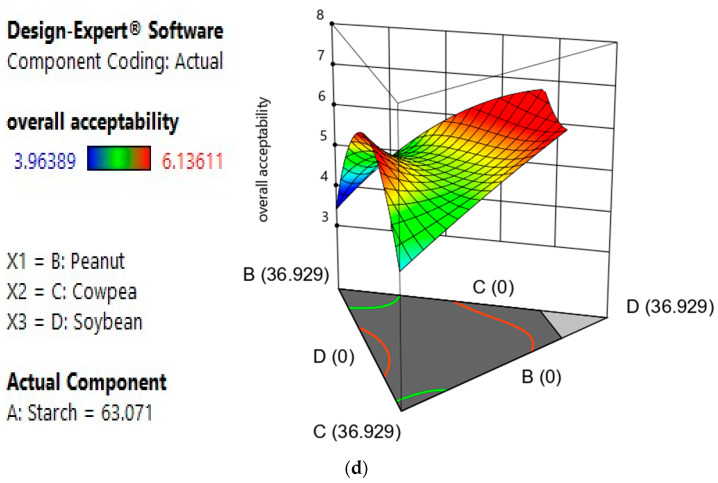
Effect of interactions of different variables on the overall acceptability. (**a**) Response surface area of A, B and C interactions on the overall acceptability; (**b**) response surface area of B, C and D interactions on the overall acceptability; (**c**) response surface area of A, B and D interactions on the overall acceptability; (**d**) response surface area of A, D and C interactions on the overall acceptability.

Sensory analysis includes all the sensations that a person may experience when holding or eating food. It is also used to determine the texture of foods [37]. Hedonic analysis in particular is a tool used when determining the acceptability of a product in a given population [38]. In this work, hedonic analysis allowed us to determine the acceptability of bread with the incorporation of different protein sources. The results of the analysis of variances (Table 5) show that cassava starch (A), peanut flour (B), cowpea flour (C), soybean flour (D) and blends of peanut–cowpea (BC) and peanut–cowpea–soybean (BCD) flour are significant model terms. The special cubic model (Table 5) was found to be the best model for overall acceptability, as it had a low PRESS value, high R^2^ and high adjusted R^2^ [26]. Concerning the response surface analysis (Figure 1), it can be highlighted that cassava starch, peanut and cowpea have a positive effect on the acceptability of bread at the intervals of 46–86%, 0–40% and 0–18%, respectively. However, soybean has a negative effect from 2% and above and a positive effect on the response in the absence of peanut or cowpea. Moreover, the regression equation (Y_1_) shows that cassava starch (A), peanut flour (B), soybean flour (D) and flour blends (starch–peanut, starch–peanut, starch–cowpea, peanut–cowpea, peanut–soybean, cowpea–soybean and starch–peanut–soybean) have a positive influence on the overall acceptability, while cowpea flour (C) and flour blends (starch–peanut, starch–soybean, peanut–soybean, starch–peanut–soybean and peanut–cowpea–soybean) influence the response negatively.

Y_1_ = 4.03A + 3.41B − 10.62C + 15.83D − 1.59AB + 25.12AC − 6.84AD + 149.19BC − 95.94BD + 6.04CD − 125.01ABC + 203.5ABD − 3.74ACD − 576.53BCD

Generally, the incorporation of legume flour in gluten-free bread results in an improvement of the overall acceptability of the product associated with improved scores for textural characteristics and appearance. In this study, we observed an improvement in the appearance and textural characteristics with the incorporation of legumes from 3.9 to 6.45 and 4.13 to 6.13, respectively, on a 9-point scale. The high protein content of legume flours enables them to improve the texture and the color of the bread [39], which results in better overall acceptability. Indeed, when proteins associate with starch molecules, they form a network capable of not only retaining the water vapor bubbles produced during baking but also expelling them once the expansion limit is reached [40]. This prevents the undesirable contraction of starch that often occurs at the end of baking, caused by excess gelatinization, resulting in a crusty texture of the bread [4]. Legumes have water-binding, oil-binding, emulsifying and foaming properties, which could contribute to improving the quality of the product in which it is incorporated, especially in small amounts [41]. These proteins also contribute to the formation of non-enzymatic browning or Maillard reaction products responsible for the color development, flavor and aroma of bread [42]. Indeed, the interactions between legume proteins, polysaccharides and volatile compounds modify the taste and smell of products enriched with legumes [43]. Lipid degradation and fatty acid oxidation produce undesirable flavors due to the activation of endogenous lipoxygenases that catalyze the oxidation of polyunsaturated fatty acids [43], which results in an unpleasant odor called bean factor, responsible for the aroma and unpleasant flavor that limits the use of legumes in food formulation [44]. These results were also observed by Dankwa et al. [45] in their study on the sensory profiles of flatbreads made from sorghum, cassava and cowpea flour, where at high percentages of incorporation of cowpea flour, panelists perceived a beany flavor.

#### 3.2.4. Effect of Components, Model Fitness, Model Testing and Responses Surface Analysis of Bread’s Specific Volume

**Figure 2:**
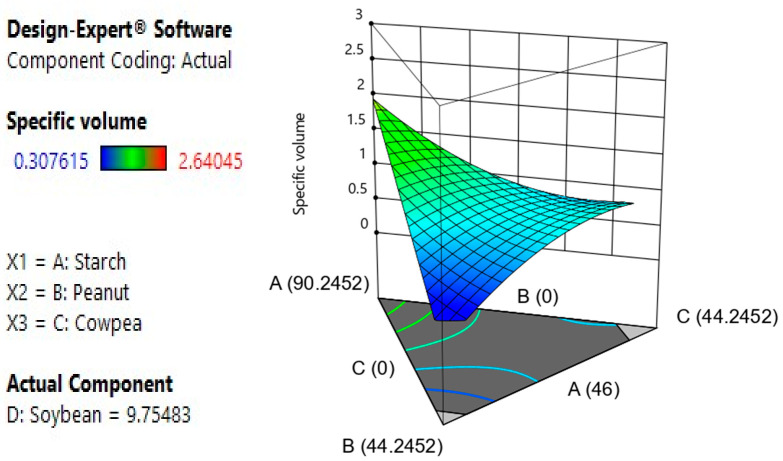
Effect of the interactions of the different variables on the specific volume. Response surface area of the interactions between starch, peanut and cowpea on the specific volume.

Specific volume is an important measure of bakery product performance. It is a measure of volumetric expansion, which is the sum of radial and axial expansions [10]. Specific volume is one of the most important indicators of bread’s technological quality, as it strongly influences consumer choice. It is used to express the technological suitability of a bread formulation [46]. The results of the analysis of variance show that A and AD are significant model terms. The quadratic model (Table 5) was found to be the best model for specific volume, as it had a low PRESS value, high R^2^ and high adjusted R^2^ [26]. The response surface analysis (Figure 2) shows the effect of factors and interactions between factors on the specific volume of bread. It appears that when soybean is maintained (9.75), peanut and cowpea negatively influence the volume of bread and starch positively influences the volume. Moreover, the regression equation (Y_2_) shows that there is a positive individual effect of starch, cowpea and soybean flour, and a quadratic effect of flour blends (starch–peanut and peanut–cowpea) on bread volume. Also, we observe a negative individual effect of peanut flour and a quadratic negative effect of flour mixtures (starch–cowpea, starch–soy, peanut–soy, cowpea–soy) on bread volume.

Y_2_ = 2.97A − 2.38B + 3.99C + 10.90D + 0.16AB − 9.989AC − 20.45AD + 8.90BC − 10.85BD − 8.86CD

High incorporation of legume flours results in an inhibition of the expansion that originates, on the one hand, from the competition for water molecules between starch and legume flours; on the other hand, we witness a disintegration of the network formed by the starch and protein molecules due to the presence of other molecules or an imbalance of the different components [47]. Indeed, large bubbles that destroy the crumb structure can lead to a higher specific volume. Legume proteins, as a water-binding factor with stabilizing properties that are not affected by baking, can modify this effect by preventing bubble fusion in the crumb [48]. These results were also observed by Lemos et al. [31], who reported that the specific volume of bread with sour cassava starch and cheese decreased with the incorporation of amaranth flour. Similarly, Cavalcante et al. [49] observed a reduction in bread volume with the incorporation of cowpea flour in sour cheese-based bread.

#### 3.2.5. Effect of Components, Model Fitness, Model Testing and Responses Surface Analysis of Bread’s Protein Content

**Figure 3:**
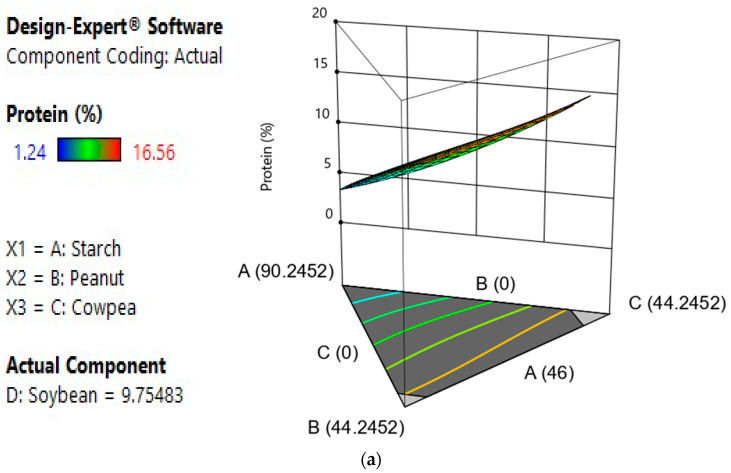

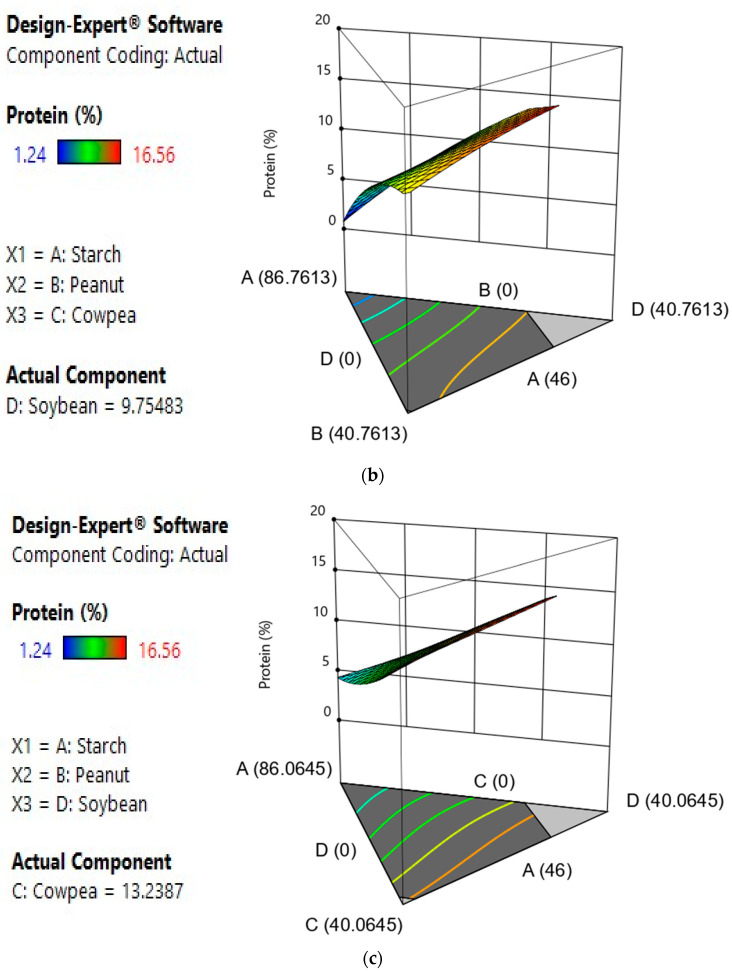

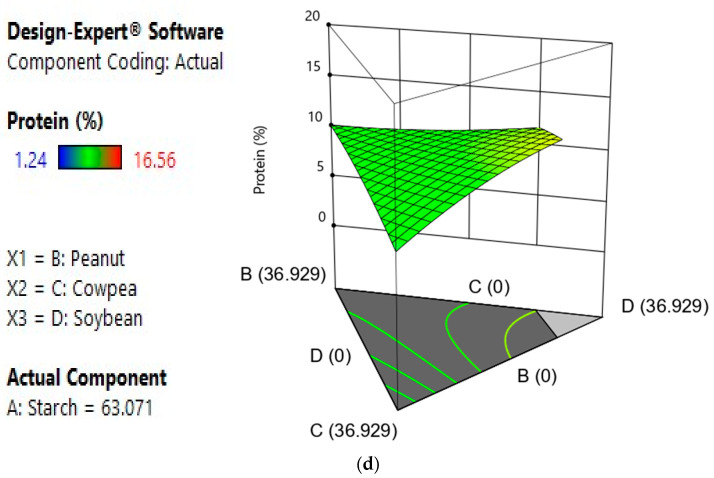
Effect of interactions of different variables on the protein content. (**a**) Response surface area of A, B and C interactions on the protein content; (**b**) response surface area of B, C and D interactions on the protein content; (**c**) response surface area of A, B and D interactions on the protein content; (**d**) response surface area of A, D and C on the protein content.

The results of the analysis of variance show that B, C and D are significant model terms, and the linear model (Table 5) was found to be the best model for overall acceptability, as it had a low PRESS value, high R^2^ and high adjusted R^2^ [26]. The response surface analysis (Figure 3) shows the effect of factors and interactions between factors on protein content. It appears that peanut, cowpea and soy positively influence response while cowpea had a negative effect on response. Moreover, the regression equation (Y_3_) shows, on one hand, that there was a positive individual effect of peanut, cowpea and soybean flours and a negative quadratic effect of starch–peanut, starch–soybean, cowpea–soybean and starch–peanut–cowpea flours. On the other hand, there was a negative individual effect of starch and a positive quadratic effect blend flour of starch–cowpea, peanut–cowpea and cowpea–soybean.

Y3 = −0.46A + 11.65B + 54.43C + 26.20D + 26.52AB − 52.44AC + 14.08AD − 102.16BC + 115.16BD − 122.63CD + 188.91ABC − 211.88ABD + 259.19ACD + 13.51BCD

Legumes are well known for their high protein content. The results of the analysis show that the protein content of peanut (28.8%), cowpea (26.8/100) and soybean (33.65%) was greater than that of cassava starch, which does not contain any protein. Thus, their incorporation in the formulation increases the bread’s protein content, as demonstrated by Lemos et al. [31], Cavalcante et al. [49] and Bonku et al. [50]. This increase in protein content is advantageous in that it will not only improve the nutritional quality of the bread but also its technological properties, such as texture and viscoelasticity [51]. Indeed, legume proteins are important for the proper functioning of the body, as they provide amino acids and carbon skeleton for the metabolic needs of the body [6]. Legume proteins are also associated with a reduction in serum cholesterol levels [52]. In addition, legume protein increases the functional and rheological properties of foods [51].

#### 3.2.6. Multiple Optimizations for Bread Formulation

**Figure 4:**
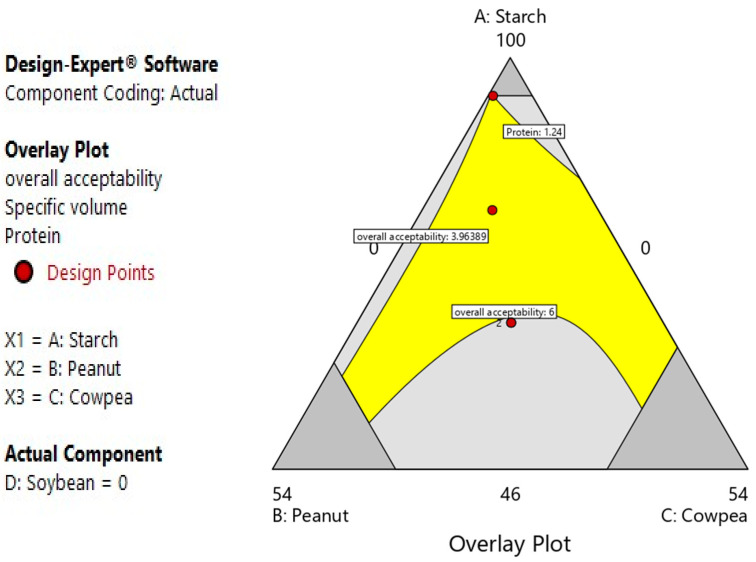
Overall plot of the optimal bread.

A compromise was made between the different optimal conditions obtained for each response, and this was used to obtain the optimal bread (Figure 4). The conditions were as follows: 64.11/18.92/16.96 for sour cassava starch, peanut flour and cowpea flour, respectively. These conditions gave the recorded experiment responses in Table 6. It appears from this table that there is no significant difference between the experimental values and the predicted values. This again confirms the validity of the model.

### 3.3. Pasting Properties of Sour Cassava Starch, Cowpea Flour, Peanut Powder and Optimum Flour

**Figure 5:**
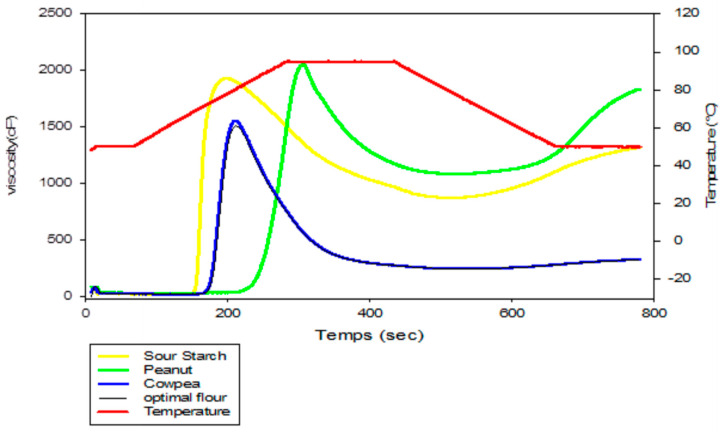
Visco-amylogram of sour cassava starch, cowpea flour, peanut powder and optimum flour.

To understand the effect of incorporating protein sources on the gelatinization and retrogradation process of starch and the relationship of these properties with the quality of our optimal bread, an analysis of RVA parameters was carried out on sour cassava starch, cowpea flour, peanut powder and optimum flour. Figure 5 shows the visco-amylogram of sour cassava starch, cowpea flour, peanut powder and optimum flour, and from this figure, we can see an increase in viscosity to a maximum, followed by a decrease to a minimum when the granules break. The viscosity then increases again, reaching a final value as it decreases. We have observed a decrease in sticking parameters with the incorporation of protein sources, which nevertheless have high viscosity compared to sour cassava starch. The reduction in viscosity with the incorporation of protein sources can be explained by the fact that other components apart from starch are abundant, particularly lipids and proteins, which significantly influence starch swelling and pasting properties [53]. These results corroborate those of Mohammed et al. [54], who found that the incorporation of chickpea flour limited the swelling of wheat flour and reduced its viscosity. Another possible explanation is that the high lipid content (around 40%) of peanut flour reduced the viscosity through the formation of lipid–amylose complexes [55]. This decrease in viscosity may also be due to a reduction in the crystallinity of the mixture caused by the presence of proteins [56]. Indeed, the proteins adhere to the starch granules and a net negative charge predominates on the surface, preventing water molecules from reaching the starch granules and thus delaying starch granule swelling [57], resulting in a lower specific volume.

Table 7 shows the RVA parameters for sour cassava starch, cowpea flour, peanut flour and the optimum flour. It is revealed that peak viscosity (PV), minimum viscosity (MV), breakdown (BD), final viscosity (FV) and setback (SB) differ significantly (*p* ˂ 0.05) for sour cassava starch, cowpea flour, peanut flour and the optimum flour. According to Singh et al. [53], there is a negative correlation between protein content and gelatinization temperature. BD is negatively influenced by protein content because proteins constitute a protective barrier against BD, and the lower the BD, the greater the resistance to shear thinning [54]. Concerning setback, which characterizes the restoration of viscosity, it increases with the incorporation of protein sources, which would mean that proteins decrease the retrogradation capacity of starch, resulting in a reduction in the hardness of the bread and being a positive factor for a long duration of bread resistance to rancidity. However, there is no significant difference (*p* ˂ 0.05) in peak time (PT) and pasting temperature (PT °C) between legume flours and optimum flour; meanwhile. there is a significant difference (*p* ˂ 0.05) between these flours and starch for PT °C. For PV, MV and FV, peanut flour has the highest value, followed by starch and optimum flour, and cowpea flour was the lowest; for BD and SB, cowpea flour had the highest value, followed by optimum flour, and peanut flour had the lowest. Indeed, the increase in pasting temperature may be due to the greater resistance of peanut and cowpea starch to swelling and breakdown. It has been reported that legume starches in general exhibit type C viscosity (restricted swelling) [58]. These results are in agreement with those of Balasubramanian et al. [59], who indicated that there was a tendency for the degree of gelatinization to reduce with increasing levels of legume incorporation.

### 3.4. Comparative Study of the Optimal Bread with Cheese Bread

#### 3.4.1. Approximate Chemical Composition

Table 8 and Table 9 show the chemical composition of cheese bread and the optimum bread. It can be highlighted that for all the parameters evaluated, the samples differ significantly. While the optimal bread had higher values of carbohydrates (67.89 g/100), fiber (2.10 g/100), Mg (69.22 mg/100 g), K (384.95 mg/100 g), Na (216.10 mg/100 g), Fe (4.43 mg/100 g), Mo (0.11 mg/100 g), Ba (0.74 mg/100 g), Cu (0.3 mg/100 g), Mn (0.66 mg/100 g) and Ni (0.75 mg/100 g), cheese bread had higher protein (10.91%), fat (10.71%), ash (2.10%), Ca (676.28 mg/100 g), P (395 mg/100 g), Si (26.15 mg/100 g), Al (3.99 mg/100 g), Ti (0.26 mg/100 g) and Zn (2.52 mg/100 g) contents. The difference is due to the incorporation of different components, namely peanut and cowpea for the optimum bread and cheese for the cheese bread (Table 8 and Table 9). It appears from these results that the substitution of cassava starch with legume flour or cheese improves its nutritional quality. This is because legumes are of higher nutritional quality than cassava starch [31]. Thus, peanut and cowpea flour are increasingly being used to improve the nutritional quality of bread, as was the case for Salve et al. [14], who demonstrated that the incorporation of peanut flour in wheat flat bread improved its nutritional properties, and Olopade et al. [60] demonstrated that the incorporation of cowpea flour in wheat bread improved its nutritional quality. The cheese bread is higher than our optimal bread; this can be explained by the fact that the rate of incorporation of cheese (54%) in the formulation is higher than that of legumes (26%). In addition, cheese is a dairy product and is generally rich in milk casein, lipids and minerals, materialized here by high ash content [61]. It is also richer in Ca, P and Na than legumes [61]. On the other hand, legumes are richer in carbohydrates, K, Fe, Zn and Cu, which explains the results obtained in the optimum bread [31]. However, the optimal bread and cheese bread are both of good nutritional quality and superior to wheat bread.

#### 3.4.2. Sensory Profile

**Figure 6:**
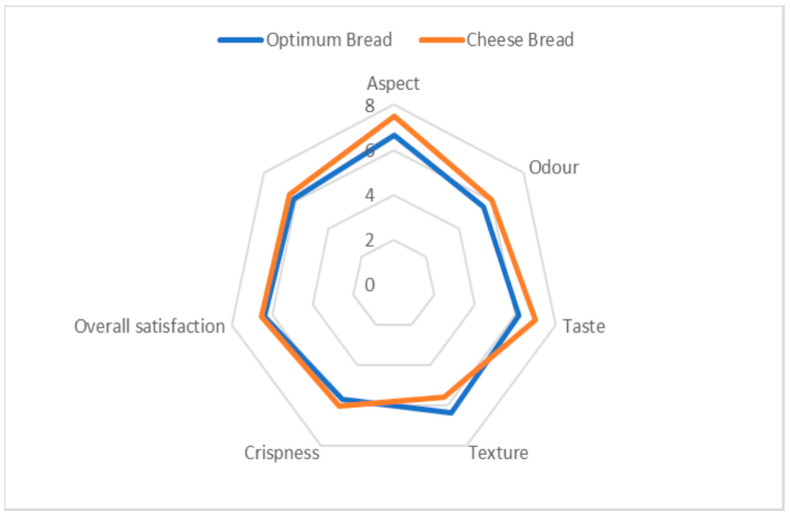
Sensorial profile of the optimum bread and cheese bread.

The optimal bread scored above 5 on a scale of 9 for all sensory attributes, which means that it was acceptable. We noted a slight superiority of the cheese bread compared to the optimal bread for aspect, odor and taste, while the optimal bread had a better score in texture than cheese bread (Figure 6). Indeed, the incorporation of leguminous flour has a less interesting impact on the appearance and taste compared to cheese bread. It leads to the darkening of the bread because of its intrinsic color and also contains simultaneously abundant carbohydrates and protein, which makes more likely the realization of the Maillard reaction, resulting in a darkening of the bread. Lemos et al. [31] also observed the blackening of cheese bread with the incorporation of amaranth flour. The products of the Maillard reaction can also have a significant impact on the modification of the aroma and flavor that limits the incorporation rate of legume flours. However, the optimal bread is superior to cheese bread for its texture. Indeed, legume proteins are known for their forming properties, and their use in food formulation increases the textural properties of the product [41]. Thus, the contribution of protein in the mixture leads to a significant modification of aroma and flavor.

#### 3.4.3. Textural Profile

Concerning the textural profile, the optimal bread was superior compared to the cheese bread in terms of hardness (40.5 N), consistency (27.30 N), elasticity (5.55 1/L) and plasticity, while the cheese bread had a better score in cohesion (0.9 N) than the optimal bread (Table 10). The globular proteins contained in legume flours have gel-forming properties; they are able to form gels by heating. Indeed, the heating leads to an exposure of hydrophobic groups within the protein, facilitating the formation of a three-dimensional network [62]. The latter results in a change in properties. The work of Olojede et al. [39] on the effect of the incorporation of legume flours on the physical and sensory properties of bread based on sorghum and sourdough showed that the presence of proteins in bread gives it a more aerated and spongy texture, which is an important criterion of bread quality. Similarly, Miñarro et al. [47], in their evaluation of the effect of legume flours on the baking characteristics of gluten-free bread, showed that bread made with chickpea flour had the most tender crumb structure.

## 4. Conclusions

This study demonstrates that peanut and cowpea flour positively influence the global acceptability of bread at moderate incorporation rates, while soybean has a negative influence even at low incorporation rates when combined with cowpea and peanut flours. The incorporation of the three legumes has a negative influence on the bread’s specific volume and a positive influence on the protein content. The optimal proportions of sour cassava starch, cowpea, soybean and peanut flour to yield acceptable bread with better nutritional and technological qualities were 64.11%, 18.92%, 0% and 16.96%, respectively. These conditions result in a desirability of 1, specific volume of 1.35 cm^3^/g, overall acceptability of 6.13, protein content of 9.72%, carbohydrate content of 67.89%, lipid content of 9.39%, fiber content of 2.10%, ash content of 1.04%, hardness of 40.5 N, cohesion of 0.70 N, consistency of 27.30 N, elasticity of 5.55 1/L, plasticity of 183.64 mj and a decrease in RVA parameters. This finding suggests that cowpea and peanut can be used for the improvement of the technological, nutritional and sensory properties of sour cassava starch bread and thus increase its consumption and application in the food processing industry, especially in developing countries where wheat is not available.

## Figures and Tables

**Table 1:** Chemical composition of cassava sour starch, peanut, cowpea and soy flour.

Sample	Starch	Peanut	Cowpea	Soybean
Protein (g/100 g)	00 ± 00 ^e^	28.8 ± 0.14 ^b^	26.87 ± 0.14 ^c^	36.02 ± 0.24 ^c^
Lipid (g/100 g)	00 ± 00 ^e^	48.82 ± 0.005 ^a^	3.09 ± 0.60 ^d^	20.02 ± 0.06 ^d^
Carbohydrate (g/100 g)	84.8 ± 1.57 ^a^	23.2 ± 0.09 ^e^	59.05 ± 0.49 ^c^	23.01 ± 0.04 ^c^
Water (g/100 g)	15.2 ± 0.03 ^a^	13.36 ± 0.04 ^a^	12.4 ± 0.035 ^b^	12.01 ± 0.35 ^b^
Fiber (g/100 g)	0.10 ± 0.05 ^a^	5.84 ± 0.04 ^b^	6.16 ± 0.02 ^a^	5.04 ± 0.08 ^a^
Ash (g/100 g)	0.70 ± 0.70 ^e^	2.65 ± 0.07 ^a^	0.75 ± 0.07 ^e^	4.09 ± 0.007 ^e^

Means in the same row with the same letters are not significantly different at the *p* < 0.05 probability level.

**Table 2:** Mineral composition of sour cassava starch, peanut powder, cowpea and soybean flour.

Sample	Starch	Cowpea	Peanut	Soybean
P (mg/100 g)	16.59 ± 1.55 ^d^	352.32 ± 2.6 ^b^	330.70 ± 1.6 ^c^	695.20 ± 10.1 ^a^
Si (mg/100 g)	27.23 ± 025 ^a^	24.32 ± 0.2 ^c^	24.80 ± 00.19 ^b^	24.2 ± 0.2 ^d^
Mg (mg/100 g)	8.08 ± 2.7 ^d^	150.27 ± 1.7 ^c^	196.91 ± 1.3 ^b^	258.24 ± 2.6 ^a^
K (mg/100 g)	66.38 ± 1.82 ^a^	909.31 ± 51.83 ^a^	820.36 ± 43.63 ^b^	1797 ± 18.9 ^a^
Ca (mg/100 g)	12.55 ± 2.04 ^d^	55.07 ± 5.78 ^b^	46.22 ± 7.48 ^c^	300.36 ± 1.6 ^a^
B (mg/100 g)	0.02 ± 0.00 ^e^	0.33 ± 0.001 ^b^	0.59 ± 0.003 ^a^	0.29 ± 0.001 ^c^
Na (mg/100 g)	1.45 ± 0.03 ^d^	4.08 ± 0.7 ^b^	2.60 ± 0.5 ^c^	3.0 ± 1.0 ^a^
Mg (mg/100 g)	7.95 ± 0.9 ^d^	141.04 ± 1.6 ^c^	182.47 ± 1.1 ^a^	152.05 ± 1.6 ^b^
Al (mg/100 g)	4.19 ± 0.05 ^b^	0.48 ± 0.09 ^d^	1.25 ± 0.02 ^c^	17.5 ± 1.2 ^a^
Ti (mg/100 g)	0.12 ± 0.003	0.06 ± 0.00	0.097 ± 0.01	
Cr (mg/100 g)	-	-	0.014 ± 0.00	0.125 ± 0.09 a
Mn	0.10 ± 0.001 ^d^	1.62 ± 0.3 ^c^	1.47 ± 0.04 ^b^	25.0 ± 0.2 ^a^
Fe (mg/100 g)	2.83 ± 1.1 ^c^	8.43 ± 0.9 ^b^	2.12 ± 1.00 ^d^	16.4 ± 1.3 ^a^
Ni (mg/100 g)	-	0.15 ± 0.07 ^a^	0.09 ± 0.001 ^c^	0.1 ± 0.001 ^b^
Cu (mg/100 g)	0.038 ± 0.0005 ^c^	0.56 ± 0.00 ^c^	1.16 ± 0.08 ^b^	14.1 ± 0.2 ^a^
Zn (mg/100 g)	0.10 ± 0.00 ^d^	4.23 ± 0.9 ^a^	3.03 ± 0.5 ^b^	2.7 ± 0.6 ^c^
Mo (mg/100 g)	-	0.34 ± 0.012 ^a^	0.23 ± 0.06 ^c^	0.24 ± 0.012 ^b^
Ba (mg/100 g)	0.042 ± 0.001 ^d^	0.60 ± 0.00 ^c^	0.63 ± 0.0005 ^a^	0.61 ± 0.00 ^b^

Means in the same row with the same letters are not significantly different at the *p* < 0.05 probability level.

**Table 3:** Anti-nutrient composition of peanut, cowpea and soy flour.

Sample	Phytate	Oxalates	Tannins
Peanut (mg/100 g)	58.67 ± 0.02 ^a^	54.07 ± 1.59 ^a^	15.28 ± 2.93 ^a^
Cowpea (mg/100 g)	45.76 ± 0.08 ^b^	43.58 ± 4.77 ^b^	13.65 ± 3.54 ^b^
Soybean (mg/100 g)	40.66 ± 0.07 ^c^	39.67 ± 4.08 ^c^	12.78 ± 2.84 ^c^

Means in the same row with the same letters are not significantly different at the *p* < 0.05 probability level.

**Table 4:** Factors and corresponding responses.

Tests Component for a 100 g Mixture					Responses		
No	X1 (g)	X2 (g)	X3 (g)	X4 (g)	Overall Acceptability (/9)	Specific Volume (cm^3^/g)	Protein Content (% g/g)
1	72.0408	0	16.3447	11.6146	5.28611	1.11105	7.35
2	65.2364	17.2673	17.4963	0	6.13611	1.26671	8.82
3	65.2364	17.2673	17.4963	0	6.03333	1.22783	8.92
4	46	20.0392	24.3225	9.63828	5.60278	0.9446	14.67
5	72.0408	0	16.3447	11.6146	5.62222	1.10165	8.3
6	52.5438	40	7.26479	0.191413	5.63278	0.74947	11.92
7	55.4307	9.86142	9.54213	25.1657	5.79444	0.434035	13.83
8	56.8757	3.77427	38	1.35	4.98889	1.07176	10.76
9	51.8986	26.8735	9.26763	11.9602	5.15278	0.92716	13.35
10	84.2554	1.26363	0	14.481	5.08333	1.31816	5.44
11	66.8888	22.4261	0	10.6851	5.34722	1.02632	9.4
12	66.8888	22.4261	0	10.6851	5.42778	0.82256	9.5
13	46	0	26.3752	27.6248	5.91667	1.08333	16.56
14	58.0867	0.795395	26.4669	14.651	5.30278	0.51182	12.42
15	52.5438	40	7.26479	0.191413	5.925	0.74947	11.94
16	95	4.45294	0.547063	0	3.96389	2.64045	1.24
17	46	20.0392	24.3225	9.63828	5.73333	0.95646	14.67
18	79.9959	12.0299	7.97417	0	4.75	2.1401	5.05
19	46	27.35	0	26.65	5.81111	0.307615	16.31
20	71.9847	0.015275	0	28	5.91389	1.27975	9.84

X1 = quantity of sour starch, X2 = quantity of peanut, X3 = quantity of cowpea, X4 = quantity of soybean.

**Table 5:** Comparison of different RSM models for overall acceptability, specific volume and protein content.

Responses	Model	*p*-Value	Lack of Fit *p*-Value	R^2^	R^2^ Adj	R^2^ Pred	PRESS	Remark
Acceptability	**linear**	**0.0304**	**0.0079**	**0.4182**	**0.3091**	**0.0470**	**4.83**	**Suggested**
	quadratic	0.0395	0.0247	0.8121	0.6431	−1.0006	10.14	**Suggested**
	**Special cubic**	**0.0100**	**0.4174**	**0.9735**	**0.9161**	**−4.1082**	**25.90**	
	cubic	0.4174		0.9771	0.9129			Aliased
Specific volume	linear	0.0004	0.0005	0.6729	0.6116	0.4315	3.07	
	**Quadratic**	**0.0272**	**0.0018**	**0.9031**	**0.8160**	**0.4451**	**2.99**	**Suggested**
	Special cubic	0.0978	0.0022	0.9692	0.9025	−36.5607	202.63	
	Cubic	0.0022		0.9960	0.9848			Aliased
Protein content	**linear**	**<0.0001**	**0.8237**	**0.9966**	**0.9960**	**0.9953**	**1.40**	**Suggested**
	Quadratic	0.8257	0.6475	0.9974	0.9950	0.9861	4.10	
	Special cubic	0.4553	0.9713	0.9984	0.9951	0.9932	2.02	
	**cubic**	**0.9713**		**0.9984**	**0.9941**			Aliased

**Table 6:** Predicted and experimental responses obtained under the same conditions for the optimal formulation.

Responses	Predicted Optimal Values	Experimental Optimal Values	Desirability
Acceptability	6.18 ^a^	6.13 ± 0.016 ^a^	1
Specific volume	1.35 ^a^	1.34 ± 0.016 ^a^	
Protein content	9.26 ^a^	9.3 ± 0.186 ^a^	

Means in the same row with the same letters are not significantly different at the *p* < 0.05 probability level.

**Table 7:** Pasting properties of sour cassava starch, cowpea, peanut and optimum flour.

Parameters	Starch	Peanut	Cowpea	Optimum
PV (Cp)	1930 ± 15.48 ^b^	2049 ± 72.53 ^a^	1552 ± 8.48 ^c^	1509 ± 7.46 ^d^
MV (Cp)	972 ± 28.44 ^b^	1180 ± 82.02 ^a^	275.5 ± 4.94 ^c^	273 ± 4.74 ^d^
BD (Cp)	958 ± 44.24 ^c^	869 ± 40.50 ^d^	1276.5 ± 13.43 ^a^	1236 ± 9.43 ^b^
FV (Cp)	1315.5 ± 36.88 ^b^	1830 ± 45.66 ^a^	324.5 ± 4.95 ^c^	321 ± 2.94 ^d^
SB (Cp)	614.5 ± 52 ^c^	219 ± 26.87 ^d^	1227.5 ± 13.45 ^a^	1188 ± 12.43 ^b^
PT (S)	780 ± 0.00 ^a^	780 ± 0.04 ^a^	780 ± 2.82 ^a^	780 ± 0.00 ^a^
PT °C (Cp)	49. 3 ± 0.00 ^b^	50.45 ± 0.00 ^a^	50.04 ± 0.007 ^a^	50.4 ± 0.00 ^a^

Means in the same row with the same letters are not significantly different at the *p* < 0.05 probability level. PV: peak viscosity, MV: minimum viscosity BD: breakdown, FV: final viscosity, SB: setback, PT: peak time, PT °C: pasting temperature, CP: centipoise, S: second.

**Table 8:** Approximate chemical composition of cheese bread and the optimum bread.

Sample	Cheese Bread	Optimum Bread
Protein (g/100 g)	10.91 ± 0.45 ^a^	9.72 ± 0.54 ^b^
Fat (g/100 g)	10.71 ± 1.17 ^a^	9.39 ± 0.49 ^b^
Carbohydrate (g/100 g)	39.72 ± 0.8 ^b^	67.89 ± 2.04 ^a^
Fibber (g/100 g)	0.05 ± 0.00 ^b^	2.10 ± 0.035 ^a^
Ash (g/100 g)	2.10 ± 0.035 ^a^	1.04 ± 0.04 ^b^

Results with the same letters in the same row no significant difference at the 0.05 level.

**Table 9:** Mineral profile of cheese bread and the optimum bread.

Sample	Cheese Bread	Optimum Bread
P	395± 3.77 ^a^	131.05 ± 0.50 ^b^
Si	26.15 ± 5.59 ^a^	26.61 ± 0.09 ^a^
Mg	27.31 ± 0.39 ^b^	69.22 ± 1.3 ^a^
K	103.06 ± 8.32 ^b^	384.95 ± 9.69 ^a^
Ca	676.28 ± 80.44 ^a^	42.21 ± 1.82 ^b^
B	0.02 ± 0.21 ^a^	0.19 ± 0.007 ^a^
Na	159.36 ± 0.46 ^b^	216.10 ± 0.08 ^a^
Mg	26.80 ± 0.33 ^b^	66 ± 1.77 ^a^
Al	3.99 ± 0.32 ^a^	3.18 ± 0.37 ^b^
K	93.93 ± 0.87 ^b^	364.29 ± 12 ^a^
Ti	0.26 ± 0.04 ^a^	0.17 ± 5.6 ^b^
Cr	0.027 ± 0.00 ^b^	0.03 ± 0.02 ^b^
Mn	0.06 ± 0.00 ^b^	0.66 ± 0.005 ^a^
Fe	2.50 ± 0.25 ^b^	4.43 ± 0.403 ^a^
Ni	- ^b^	0.74 ± 0.007 ^a^
Cu	0.04 ± 0.048 ^b^	0.3 ± 0.006
Zn	2.52 ± 0.0025 ^a^	1.75 ± 0.19 ^b^
Mo	- ^b^	0.11 ± 0.001 ^a^
Ba	0.04 ± 0.00 ^b^	0.27 ± 0.014 ^a^

Results with the same letters in the same row show no significant difference at the 0.05 level.

**Table 10:** Textural profile of cheese bread and the optimum bread.

Sample	Cheese Bread	Optimum Bread
Hardness	46 ± 1.0 ^a^	40.5 ± 1.5 ^b^
Cohesiveness	0.9 ± 0.2 ^a^	0.70 ± 0.3 ^b^
Consistency	38.1 ± 3.7 ^a^	27.30 ± 1.8 ^b^
Elasticity	5.24 ± 0.3 ^b^	5.55 ± 0.46 ^a^
Plasticity	185 ± 2.0 ^a^	183.64 ± 2.4 ^b^

Results with the same letters in the same row show no significant difference at the 0.05 level.

## Data Availability

The datasets generated for this study are available on request to the corresponding author.
